# Variation among Soybean Cultivars in Mesophyll Conductance and Leaf Water Use Efficiency

**DOI:** 10.3390/plants5040044

**Published:** 2016-12-11

**Authors:** James Bunce

**Affiliations:** USDA-ARS, Crop Systems and Global Change Laboratory, Beltsville, MD 20705, USA; James.Bunce@ars.usda.gov; Tel.: +1-301-504-7629

**Keywords:** soybean, water use efficiency, mesophyll conductance, stomatal conductance, internal CO_2_ concentration, photosynthesis

## Abstract

Improving water use efficiency (WUE) may prove a useful way to adapt crop species to drought. Since the recognition of the importance of mesophyll conductance to CO_2_ movement from inside stomatal pores to the sites of photosynthetic carboxylation, there has been interest in how much intraspecific variation in mesophyll conductance (g_m_) exists, and how such variation may impact leaf WUE within C_3_ species. In this study, the g_m_ and leaf WUE of fifteen cultivars of soybeans grown under controlled conditions were measured under standardized environmental conditions. Leaf WUE varied by a factor of 2.6 among the cultivars, and g_m_ varied by a factor of 8.6. However, there was no significant correlation (r = −0.047) between g_m_ and leaf WUE. Leaf WUE was linearly related to the sub-stomatal CO_2_ concentration. The value of g_m_ affected the ratio of maximum Rubisco carboxylation capacity calculated from the sub-stomatal CO_2_ concentration to that calculated from the CO_2_ concentration at the site of carboxylation. That is, variation in g_m_ affected the efficiency of Rubisco carboxylation, but not leaf WUE. Nevertheless, there is considerable scope for genetically improving soybean leaf water use efficiency.

## 1. Introduction

With increasing limitations on the amount of water available to support agriculture, increasing the water use efficiency (WUE) of crops—the ratio of crop dry mass gained to water consumed—is a reasonable objective. Selection for high leaf WUE in wheat improved yield in dry conditions [1]. Leaf WUE is often defined as the ratio of photosynthesis to transpiration. However, the rate of transpiration is directly related to the difference in water vapor pressure between the air inside and outside the leaf (the leaf to air vapor pressure difference, LAVPD), so the LAVPD during the measurement has a large impact on WUE. Because of this, comparisons of leaf WUE often use “intrinsic” water use efficiency [2], the ratio of photosynthesis to stomatal conductance.

Limitations to C_3_ photosynthesis at high light and at the current ambient CO_2_ concentration have long been quantified by the maximum capacity of rubisco carboxylation (V_Cmax_) [3]. However, it has been recognized within the last several years that mesophyll conductance to CO_2_ movement from inside the stomata to the site of fixation within chloroplasts (g_m_) can be a significant limitation to photosynthesis (A) [4,5]. Because of the potential of variation in g_m_ to affect A independently from stomatal conductance (g_s_), there is interest in determining the extent of variation in g_m_ within species, and its effect on leaf WUE.

Intraspecific variation in g_m_ has been reported in barley [6], grape [7], tomato [8], wheat [9], and rice [10], and its relationship to leaf WUE examined. In grape and barley, g_m_ was positively correlated with leaf WUE. In tomato and rice, variation was found in the ratio of g_m_ to g_s_, and that ratio was correlated with leaf WUE. In wheat variation, g_m_ was correlated with A, but there was no clear relationship between g_m_ and leaf WUE.

Among these studies, g_m_ was quantified either from leaf fluorescence changes with CO_2_ concentration, or from carbon isotope discrimination, both of which methods are based on assumptions which are sometimes dubious [11,12,13]. A more rapid method for estimating g_m_, with fewer assumptions, has been developed based on the response of photosynthesis to oxygen concentration [14], and was used here to screen fifteen cultivars of soybean for g_m_ and to test for relationships among g_m_, g_s_, A, and leaf WUE. Genetic improvement of WUE depends upon sufficient genetic variation in WUE and the identification of physiological processes which affect it, which, as indicated by the literature cited, may vary with crop species. The goals of this study were to determine whether variation in intrinsic leaf WUE or g_m_ occurred in soybean measured under standardized conditions, and to determine whether any variation in g_m_ was related to variation in intrinsic leaf WUE.

## 2. Results

**Figure 1:**
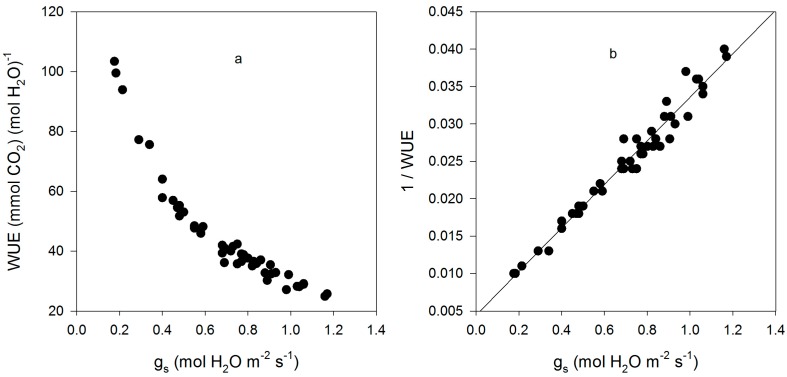
Leaf intrinsic water use efficiency (WUE) (**a**); and the reciprocal of leaf intrinsic WUE (**b**) as a function of stomatal conductance (g_s_) for all measurements made on fifteen cultivars of soybean. The linear regression of (1/WUE) = 0.0044 + 0.029 × g_s_ had an r^2^ value of 0.965.

**Figure 2:**
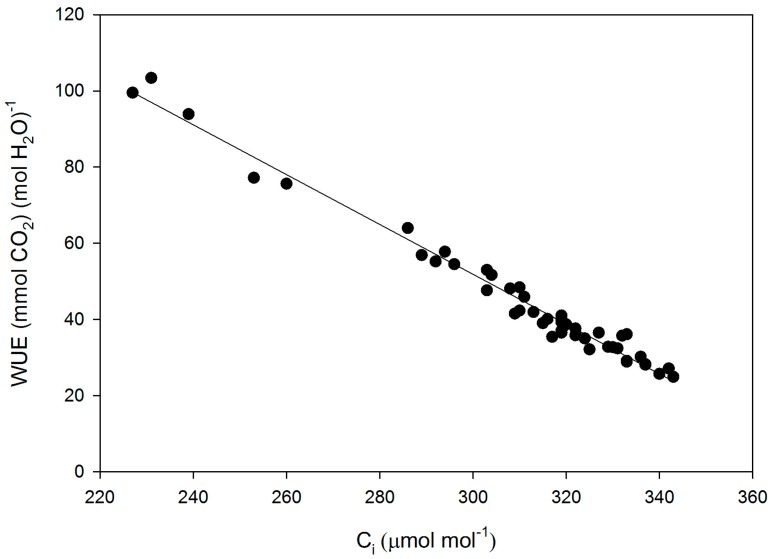
Leaf intrinsic water use efficiency (WUE) as a function of sub-stomatal CO_2_ concentration (C_i_) for all measurements made on fifteen cultivars of soybean. The linear regression: WUE = 248 − 0.654 × C_i_ had an r^2^ of 0.98.

The cultivars differed significantly in g_s_, g_m_, leaf intrinsic WUE, sub-stomatal CO_2_ concentration (C_i_), and the CO_2_ concentration at rubisco (C_c_), but not in V_Cm_C_c_ (V_Cmax_ based on C_c_), V_Cm_C_i_ (V_Cmax_ based on C_c_), or A (Table 1). For cultivars with high and low values of g_m_, the values estimated using the initial slope of A vs. C_i_ agreed with those using the O_2_ effect. Intrinsic leaf WUE varied by a factor of 2.6 among the cultivars, with Fiskeby having the highest, and Ford having the lowest WUE (Table 1). Mesophyll conductance varied by a factor of 8.6 among cultivars, with Biloxi having the highest and Clark the lowest mean values. Variation in cultivar means of intrinsic WUE was due mostly to variation in g_s_, which ranged from 0.38 to 1.12 mmol H_2_O m^−2^·s^−1^, rather than photosynthesis, which ranged from 23.7 to 31.1 μ mol CO_2_ m^−2^·s^−1^. Fiskeby, with the highest WUE, had the second lowest g_s_, and Ford, with the lowest WUE, had the highest g_s_. For all individual leaf measurements, intrinsic WUE was strongly negatively related to g_s_, with the reciprocal of WUE linearly related to g_s_ (Figure 1). C_i_ ranged from about 225 to 345 μ mol·mol^−1^ and there was a negative linear relationship between C_i_ and intrinsic WUE (Figure 2).

**Figure 3:**
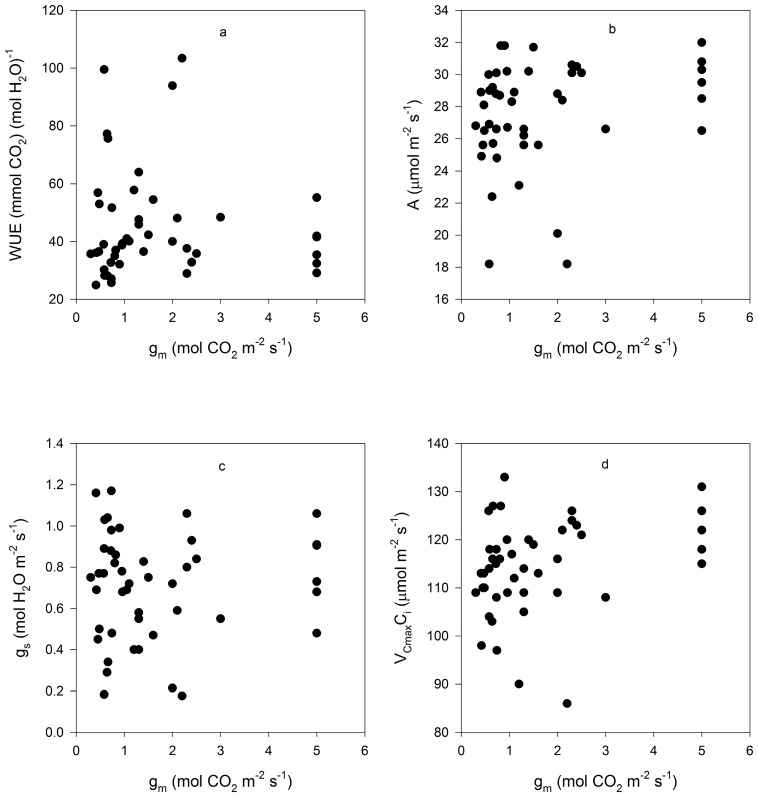
Correlations between g_m_ and (**a**) leaf intrinsic water use efficiency (WUE); (**b**) CO_2_ assimilation rate (A); (**c**) stomatal conductance (g_s_); (**d**) and the maximum rate of rubisco carboxylation based on C_i_ (V_Cmax_C_i_) for all measurements made on fifteen cultivars of soybean. Correlation coefficients were not significant at *p* = 0.05, and are given in the text.

**Figure 4:**
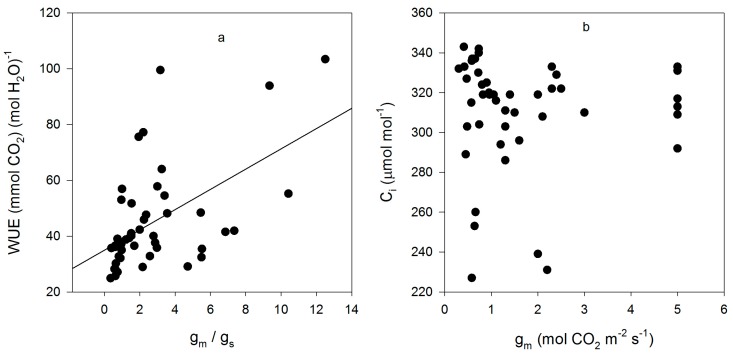
Relationships between the ratio of g_m_ to g_s_ and WUE (**a**); and between g_m_ and C_i_ (**b**) for all measurements made on fifteen cultivars of soybean. The correlation between the g_m_ to g_s_ ratio and WUE had an r value of +0.53, and that between g_m_ and C_i_ was +0.116, which was not significant at *p* = 0.05.

**Figure 5:**
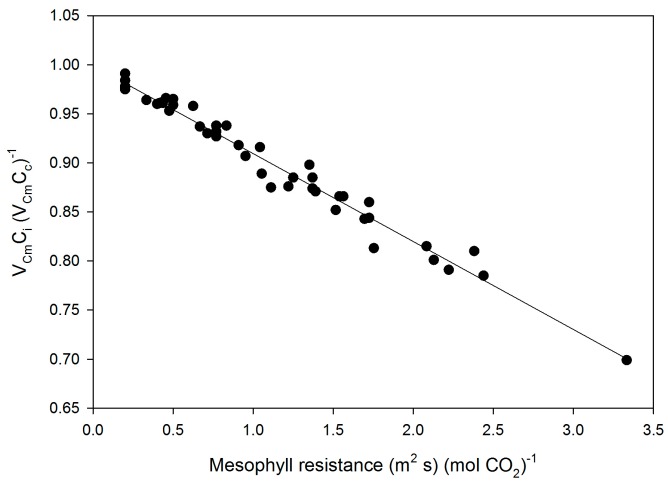
The ratio of V_Cmax_ based on C_i_ to that based on C_c_ as a function of mesophyll resistance, the reciprocal of g_m_, for all measurements made on fifteen cultivars of soybean. The linear regression: V_Cm_C_i_/V_Cm_C_c_ = 1.00 − 0.090 × (1/g_m_) had an r^2^ of 0.976.

**Figure 6:**
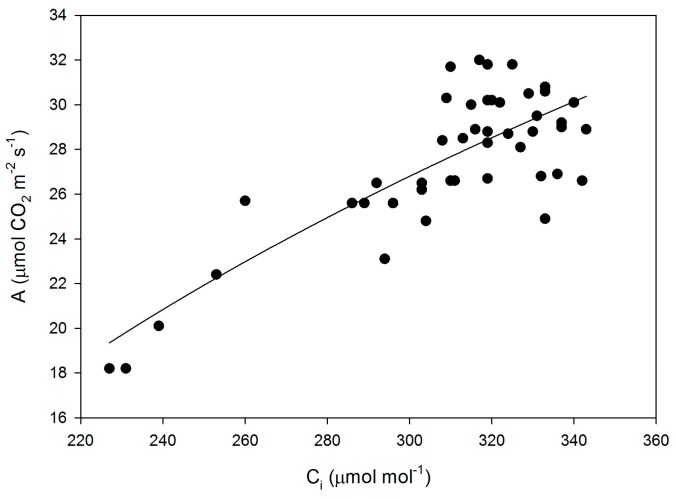
CO_2_ assimilation rate (A) as a function of sub-stomatal CO_2_ concentration (C_i_) for all measurements made on leaves of fifteen cultivars of soybean. The equation A = −125.6 + 26.7 ln(C_i_) had an r^2^ value of 0.645.

**Figure 7:**
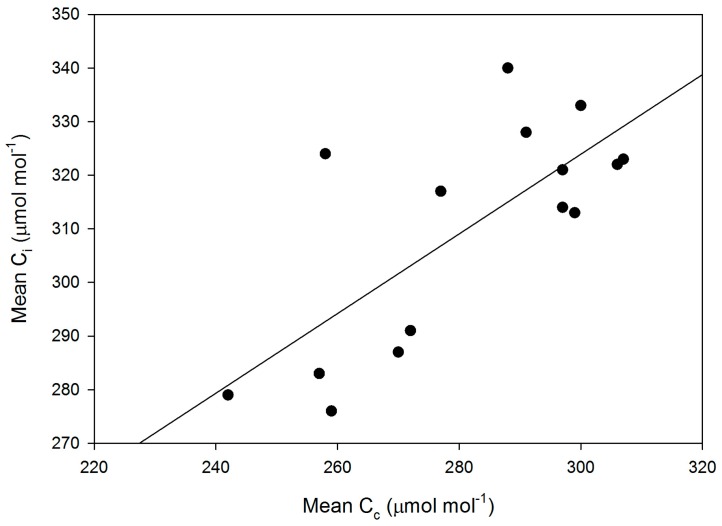
Cultivar mean values of C_i_ versus mean values of C_c_. The linear regression had an r^2^ value of 0.532.

Mesophyll conductance had no significant correlation with intrinsic WUE (r = −0.047), g_s_ (r = +0.065), A (r = +0.234), or V_Cmax_C_i_ (r = +0.227) (Figure 3). Using means for the cultivars for all of these parameters also produced no significant correlations (r = 0.079, 0.217, 0.040, and 0.301, for g_m_ versus intrinsic WUE, g_s_, A, and V_Cmax_C_i_, respectively). The ratio of g_m_ to g_s_ in soybean had a small but significant correlation with intrinsic leaf WUE (Figure 4), but there was no significant correlation between C_i_ and g_m_ (Figure 4). The ratio of the maximum capacity of rubisco carboxylation modelled based on C_i_ to that that based on C_c_ decreased with mesophyll resistance, which is the reciprocal of g_m_, from about 0.70 to 0.98 (Figure 5). Photosynthesis at C_a_ = 400 μ mol·mol^−1^ was positively related to C_i_ (Figure 6), similar to a saturating A vs. C_i_ curve for an individual leaf. While there was a small but significant correlation between C_i_ and C_c_ (Figure 7), there was a wide range of C_i_ values for a given C_c_. For example cultivar means of C_i_ ranged from about 275 to 325 μ mol·mol^−1^ for mean C_c_ values of about 256 to 259 μ mol·mol^−1^ (Figure 7).

## 3. Discussion

The linear relationship between intrinsic leaf WUE and C_i_ was as expected, since C_a_ was constant across the measurements. Carbon isotope discrimination has long been used as a surrogate for C_i_ in screening for leaf WUE in C_3_ species. It is important to know how much variation in g_m_ would disrupt the correlation between C_i_ (and WUE) and carbon isotope discrimination, which should reflect C_c_ rather than C_i_ [6]. The ranking of these soybean cultivars based on C_c_ or carbon isotope discrimination would not provide a reliable ranking of their C_i_ or intrinsic leaf WUE values (Figure 7).

While variation among cultivars in C_i_ was more strongly influenced by variation in g_s_ than A, there was still the usual [15] penalty in A associated with low g_s_, low C_i_ and high intrinsic WUE (Figure 5). One reason for interest in g_m_ with regard to WUE is the possibility that high g_m_ might offset the penalty in A associated with low C_i_ and high WUE [16]. Certainly, a high g_m_ results in a higher value of A at a given C_i_, and results in V_Cm_C_i_ being closer to V_Cm_C_c_ (Figure 4). However, g_m_ was poorly correlated with any other leaf gas exchange parameter (g_s_, A, C_i_, V_Cm_C_i_, WUE) in this data. It is possible that the correlation between the ratio of g_m_ to g_s_ and WUE reported in rice and tomato [8,10] has little to do with variation in g_m_, but simply reflects a strong relationship between g_s_ and WUE, as found here in soybean. Furthermore, it remains unknown how much a genetic increase in g_m_ might cost in terms of leaf nitrogen, which would tend to offset the resulting increased efficiency of rubisco [16]. It is thought that there is a metabolic component to g_m_, and that therefore there would be some nitrogen cost to increasing g_m_ [16]. Reasons for cultivar differences in the operational C_i_ under these identical and non-stressful environmental conditions remain unknown, but g_s_ and the operational C_i_ were clearly the primary determinates of leaf intrinsic WUE among these soybean leaves.

The observed 2.95 fold range in mean g_s_ among soybean cultivars would have a substantial impact on canopy transpiration, even though relative differences in transpiration decrease as the scale increases from leaf to canopy [17]. Using the mean leaf boundary layer conductance of 1200 mmol (H_2_O) m^−2^·s^−1^ of soybean leaves measured near midday on 9 days in Beltsville, Maryland [18], a 2.95 fold range in leaf g_s_ would translate into approximately a 1.45 fold range in canopy transpiration. Such a large range in canopy transpiration could have a substantial impact on the rate of development of plant water stress in the field. Thus, there is considerable scope for reduction in canopy transpiration rate and improvement in soybean WUE through breeding. However, the results of this study make it unlikely that screening soybeans for g_m_ values would be a profitable method of attempting to increase WUE in soybean.

It should be noted that the intrinsic leaf WUE and g_s_ values measured here were measured under a standardized LAVPD. “Intrinsic” leaf WUE is, of course, not really intrinsic to the leaf, but would be expected to vary with the measurement conditions, as A and g_s_ vary with environment. The response of g_s_ to LAVPD in soybean is known to vary among cultivars [19]. Fletcher et al. [20] have identified lines of soybeans differing in the response of whole plant transpiration rate to LAVPD, but did not provide information about either g_s_ or photosynthetic responses to LAVPD, or information on leaf WUE. Field measurements of g_s_ and A in response to LAVPD in several soybean cultivars [21] did not present data on operational C_i_.

Our results indicated that g_s_ and C_i_ were correlated with leaf intrinsic WUE, and may be useful in selecting lines with high WUE. However, the lack of correlation between g_m_ and leaf intrinsic WUE indicates that g_m_ may not be useful in ranking lines for WUE, although both factors varied among cultivars. It will be important to assess the operational C_i_ of soybean lines under a range of field conditions to determine how robust the differences in leaf intrinsic WUE found here are in the field. The results presented here also indicate that carbon isotope discrimination may not reliably identify soybean lines with differences in leaf intrinsic WUE.

## 4. Materials and Methods

Three or four plants of each of fifteen cultivars of soybean (*Glycine max* L. Merr.) obtained from the U.S. Department of Agriculture soybean germplasm collection (Table 1) were grown together in one large controlled environment chamber at the controlled environment facility of the Beltsville Agricultural Research Center, Beltsville, Maryland. All of the cultivars are adapted to the eastern United States. Some (Biloxi, Clark, Kent, PI-471938, A5959) were chosen because they had diverse responses of stomatal conductance or transpiration rate to humidity in prior studies [19,20]. The others were chosen based on my prior measurements of leaf gas exchange rates in various experiments. The chamber had 14 h of light per day at 1000 μ mol m^−2^·s^−1^ photosynthetic photon flux density (PPFD) from a combination of metal halide and high pressure sodium lamps. This gave a daily total photon flux similar to average mid-summer days at Beltsville, Maryland. The day/night air temperatures were 26/20 °C, with a dew point temperature of about 18 °C. These approximate mean conditions in Beltsville for the soybean growing season. The CO_2_ concentration was controlled at 400 μ mol·mol^−1^ during the day and 430 μ mol·mol^−1^ at night by the addition of pure CO_2_ or air scrubbed of CO_2_, under the control of a WMA-5 (PP Systems, Amesbury, MA, USA) infrared analyzer which sampled chamber air continuously. Plants were grown one per pot in 15 cm diameter plastic pots filled with vermiculite and were fertilized daily with a complete nutrient solution containing 14.5 mN nitrogen.

Leaf gas exchange measurements were conducted on fourth main stem trifoliolate leaves a few days after those leaves reached full area expansion. Leaf gas exchange measurements were made with a recently calibrated Li-6400 portable photosynthesis system (Li-Cor, Inc., Lincoln, NE, USA). Leaf temperature was controlled at 25 °C, the PPFD was 1500 μ mol m^−2^·s^−1^, and the LAVPD was maintained at 1.0 ± 0.1 kPa. Each leaf was measured under three conditions of CO_2_ and O_2_. First, the leaf was exposed to 21% O_2_, and 400 μ mol·mol^−1^ external CO_2_. The external CO_2_ was then lowered to 300 μ mol mol^−1^ at the same O_2_ concentration. Finally, the oxygen concentration was lowered to 2%, while the CO_2_ concentration remained at 300 μ mol·mol^−1^. Care was taken to ensure that gas exchange rates were steady at each condition. These measurements were made on three or four replicate leaves from different plants for each cultivar. A, g_s_, C_i_, and LAVPD were calculated by the instrument software.

Intrinsic leaf WUE was calculated separately for each leaf as A/g_s_ measured at 400 ± 5 μ mol mol^−1^ CO_2_, and 21% O_2_, at 25 °C, and at a LAVPD of 1 kPa. Mesophyll conductance was also calculated for each leaf from the gas exchange rates at 300 μ mol·mol^−1^ CO_2_ at 21% and 2% O_2_ using the method described in Bunce [14], with the calculation utility developed by Singh [22]. The method is based on the idea that the sensitivity of photosynthesis to oxygen depends on the concentration of CO_2_ at the site of Rubisco, rather than the sub-stomatal CO_2_ concentration. This method of estimating g_m_ uses the change in photosynthetic rate between two O_2_ concentrations at limiting CO_2_ concentrations to indicate the CO_2_ concentration at the site of rubisco (C_c_), using a standard biochemical C_3_ photosynthesis model [14]. This method of estimating g_m_ also provides estimates of V_Cmax_ based on C_i_ and based on C_c_. For some leaves with the highest and lowest values of g_m_, additional points on A vs. C_i_ curves at 21% O_2_ were used to calculate g_m_ using the Sharkey et al. calculation utility [23] based on the curvature of the initial slope of the A vs. C_i_ curve.

Analysis of variance was used to test for differences among cultivars in mean leaf gas exchange parameters. For both g_s_ and g_m_, homogeneity of variance was violated, so values were log transformed prior to ANOVA. Correlations among gas exchange parameters were calculated using all data on individual leaves, because of the increased statistical power compared with using mean values, given the substantial variability among leaves with cultivars. Where correlations were significant at *p* = 0.05, linear or simple non-linear regressions are presented.

## Figures and Tables

**Table 1:** Mean leaf gas exchange parameter values of the soybean cultivars tested. Parameters were determined for three or four leaves per cultivar. g_s_ is stomatal conductance in mmol (H_2_O) m^−2^·s^−1^, g_m_ is mesophyll conductance in mmol (CO_2_) m^−2^·s^−1^, A is photosynthetic rate in μ mol (CO_2_) m^−2^·s^−1^ measured at 400 μ mol·mol^−1^ CO_2_, WUE is intrinsic water use efficiency in mmol (CO_2_) per mol (H_2_O), V_Cm_C_i_ is the maximum rate of rubisco carboxylation based on C_i_, and V_Cm_C_c_ is the maximum rate of rubisco carboxylation based on C_c_. C_i_ is the sub-stomatal CO_2_ concentration, and C_c_ is the CO_2_ concentration at rubisco, in μ mol·mol^−1^. Probability of equal means from ANOVA is given, and the Tukey–Kramer honestly significant difference (HSD) is provided when the probability is <0.05.

Cultivar	g_s_	g_m_	A	WUE	V_Cm_C_i_	V_Cm_C_c_	C_i_	C_c_
A5959	703	2.63	27.7	43.1	113	118	313	299
Biloxi	803	3.60	28.9	36.4	116	123	323	307
Chief	878	2.28	31.1	35.9	127	139	321	297
Clark	677	0.42	26.6	40.3	108	138	324	258
Essex	587	1.50	26.4	56.9	114	121	291	272
Fiskeby V	418	2.56	24.4	67.6	114	122	276	259
Ford	1123	0.60	29.4	26.2	116	138	340	288
Holt	502	2.05	25.0	63.5	105	112	287	270
Kent	820	0.78	28.0	34.7	115	132	328	291
Lincoln	725	0.77	28.4	39.1	118	137	317	277
Perry	883	2.10	30.8	35.7	121	127	322	306
PI-41	430	1.09	25.8	62.0	117	130	283	257
Ripley	380	0.76	23.7	64.0	101	118	279	242
Spencer	728	2.68	29.0	41.6	118	124	314	297
Wabash	910	1.11	28.1	31.8	114	128	333	300
**Probability**	**0.001**	**0.016**	**0.081**	**0.029**	**0.097**	**0.279**	**0.019**	**0.036**
**HSD**	**592**	**2.79**		**31.1**			**56**	**65**
